# Variation in Psychiatric Hospitalisations: A Multiple-Membership Multiple-Classification Analysis

**DOI:** 10.3390/ijerph21080973

**Published:** 2024-07-25

**Authors:** Emely Ek Blæhr, Beatriz Gallo Cordoba, Niels Skipper, Rikke Søgaard

**Affiliations:** 1 DEFACTUM, Central Denmark Region, 8000 Aarhus, Denmark; 2 Department of Public Health, Aarhus University, 8000 Aarhus, Denmark; rsoegaard@health.sdu.dk; 3 Faculty of Education, Monash University, Clayton, VIC 3800, Australia; beatriz.gallocordoba@vu.edu.au; 4 Centre for International Research on Education Systems, Mitchell Institute, Victoria University, Melbourne, VIC 8001, Australia; 5 Department of Economics and Business Economics, Aarhus University, 8000 Aarhus, Denmark; nskipper@econ.au.dk; 6 Department of Clinical Research, University of Southern Denmark, 5230 Odense, Denmark

**Keywords:** medical practice variation, psychiatry, multilevel modelling

## Abstract

The complexity of variation in healthcare, particularly in mental health, remains poorly understood. However, addressing this issue presents an opportunity to opti-mise the allocation of scarce healthcare resources. To explore this, we investigated the variation in psychiatric care measured as the number of psychiatric hospitalisations. We estimated multiple-membership multiple-classification models utilising Danish register data for 64,694 individuals and their healthcare providers, including 2101 general practitioners, 146 community-based care institutions, 46 hospital departments, and 98 municipalities. This approach recognised that data are not strictly hierarchical. We found that, among individuals attending a single healthcare provider, 67.4% of the total variance in the number of hospitalisations corresponds to differences between individuals, 22.6% to differences between healthcare providers’ geographical location, 7.02% to differences between healthcare providers, and 3% to differences between the geographical locations of the individuals. Adding characteristics to the model ex-plained 68.5% of the variance at the healthcare provider geographical level, but almost no explanation of the variation was found on the three other levels despite the nu-merous characteristics considered. This suggests that medical practice may vary un-warrantedly between healthcare providers, indicating potential for optimisation. Streamlining medical practices, such as adhering to clinical guidelines, could lead to more efficient supply of mental health resources. In conclusion, understanding and addressing variation in psychiatric care may impact resource allocation and patient outcomes, ultimately leading to a more effective healthcare system.

## 1. Introduction

Globally, healthcare systems are challenged by substantial variation in service use and costs, which often indicate inefficiencies and inequities in care delivery [1]. This issue is particularly critical in fields like psychiatry, where consistent and effective treatment is essential for managing chronic mental health conditions. There is a growing demand for mental health services, and they impose a significant financial burden on national economies, accounting for 3–4% of GDP in developed countries [2]. However, there remains a gap in understanding the sources and extend of these variations within psychiatric care as the existing evidence is limited.

Research in other healthcare areas has documented that variations in medical practice are often unwarranted; arising from factors unrelated to individuals’ needs or preferences [3,4,5]. However, psychiatric care has been underexplored in this context, leaving a significant gap in both academic literature and policy-making. Given that mental illness accounts for a substantial and increasing proportion of healthcare expenditure, optimising psychiatric care delivery is crucial for both economic and public health outcomes.

Existing literature indicates that variations in psychiatric practice may be attributed to three primary factors: differences between individuals, differences between locations, and differences between healthcare providers [6,7,8,9,10]. For example, Weich et al. (2017) found that 84.7% of the variation in compulsory hospitalisations in England was due to individual differences, 8.3% to local area differences, and 7.0% to provider differences [10]. Gandré et al. (2018) observed significant local area variation in psychiatric hospitalisations and rehospitalisations in France, although their ability to explain this variation was limited by the characteristics they could include [7].

In terms of psychiatric hospitalisation length, previous studies suggest that provider-level factors, such as hospital type and case mix and individual characteristics play significant roles [8,11,12,13]. Gifford and Foster (2008) found that provider-level factors accounted for 51% of the variability in length of stay, while Crossley and Sweeney (2020) noted that individual-level factors influenced the length of each stay, and provider-level factors impacted the overall duration of stays [8,11].

The gap that this study aims to fill is the lack of a comprehensive understanding of the sources and extent of variation in psychiatric hospitalisations in a health care system like the one in Denmark [14,15]. Despite some existing studies, there is limited empirical evidence that integrates individual, provider, and regional factors within a unified analytical framework [7,10,11]. Previous research frequently encountered a deficit of detailed, multilevel data necessary to fully explain the observed variations, and it was methodologically limited. This study seeks to address this gap by leveraging comprehensive, register-based data from Denmark and employing advanced statistical models. Building on existing literature and the Andersen Behavioural Model, our study employs a multiple-membership multiple-classification (MMMC) model to decompose the variation in psychiatric hospitalisations and explore medical practice variation [7,10,16,17,18]. Unlike strictly hierarchical multilevel models, the MMMC model accommodates individuals treated by multiple healthcare providers across different municipalities, reflecting the complex nested structure of the Danish healthcare system. We also leverage comprehensive data from all individuals within the entire healthcare system, including diverse individual, healthcare provider, and local area characteristics. Jointly, this approach enhances model validity, increases the likelihood of explaining practice variation, and offers crucial insights to inform decision-making in psychiatric care.

The aim of this study is to examine the extent and sources of variation in psychiatric hospitalisations and quantify the extent to which demand-side (individual), supply-side/medical practice (HCP and community-based care provider), practice context/small-area (municipality) and their characteristics explain any variation. According to the existing literature, our hypothesis is that most of the variation is attributed to the demand-side, second-most to the practice context and the least to the supply-side.

This study contributes to the broader literature on healthcare variation by applying a novel methodological approach to a comprehensive dataset, offering a robust analysis that can be replicated in other settings and health systems. By addressing these critical questions, this study not only advances the understanding of practice variation in psychiatry but also has the potential to inform policies for optimising mental health service delivery, therefore enhancing the overall efficiency and effectiveness of the healthcare system.

### Andersen Behavioural Model of Utilisation

To comprehend the factors influencing mental health care variation, we applied the Andersen Behavioural Model of Utilisation [19]. This model states that health care utilisation is driven by predisposing factors (individual characteristics existing before illness onset), enabling factors (resources facilitating or hindering healthcare access), and need factors (driven by perceived or evaluated service necessity).

Predisposing factors include intrinsic characteristics that predispose individuals to treatment, such as demographic attributes (age, gender, nationality, marital status, and household composition) and social structural elements (labour market engagement, income levels, and exposure to crime) [20,21]. Previous health characteristics, including long-term diagnoses and psychiatric and somatic comorbidities are also relevant [20]. Our hypothesis is that these factors can affect the number of psychiatric hospitalisations by influencing health behaviours and attitudes towards seeking care.

Enabling factors encompass resources at the individual, community, and municipal levels that either facilitate or impede healthcare access and utilisation. Individual resources, such as financial means, and community resources, including healthcare provider and facility characteristics, are critical [22]. Municipal resources like assisted living facilities, support services, psychological therapy provisions, population density, and demographic composition are also relevant [23]. We hypothesise that a greater availability of resources will be associated with a higher utilisation of psychiatric services due to reduced barriers to care.

Need factors include health status indicators and service requirements. Health status is defined by diagnosis clusters, comorbid conditions, and symptoms like depressive symptoms and suicidal tendencies [20]. Service needs are assessed through healthcare utilisation metrics, such as the duration of hospital stays per admission. We hypothesize that individuals with greater need (e.g., severe mental illness) are more likely to be hospitalised for a larger number of days.

The final model component represents healthcare utilisation patterns influenced by healthcare provider (HCP) characteristics and healthcare provider municipality (HCPM) attributes [10,24].

In addition to the traditional Andersen model, we also build on Phillips et al.’s (1998) study that explored the use of environmental and provider-related variables in existing literature [25]. They found that, in their paper, roughly half of the studies using the behavioural model included environmental or provider-related variables. Most of the studies that included environmental variables measured urban/rural location or region as broad proxies for specific indicators like service supply or access to care. While many studies included some provider-related variables, such as service usage, few considered provider characteristics, such as specialty or physician gender. Phillips et al. found that most of the studies utilising explanatory methods involved hierarchical entry of variables, with other methods being rare. They concluded that this underutilisation of contextual variables and explanatory methods could lead to biased and misleading results, and that this was a key reason for the low variance typically explained in these studies. Based on this conclusion, our study properly considers the role of both provider and geographical or environmental characteristics.

## 2. Methods

### 2.1. Setting

Danish citizens have free access to a fully tax-funded healthcare system. Psychiatric care can be accessed in two ways: (1) through one of the 3443 GPs working in the public sector, or (2) through an emergency ward who can refer the individual to appropriate care [26]. The GP acts as a gatekeeper to psychiatric care when the individual’s state of illness is not acute. The individual can then be referred to three different alternatives: (1) specialised care, defined as psychiatric hospitals or psychiatric wards in bigger hospitals who supply inpatient treatment; (2) psychiatric community care, defined as community-based care and outreaching teams who supply outpatient treatment closer to or in the individual’s home; or (3) a private psychiatrist. If the individual is referred to any of these treatment alternatives, they will not have any out-of-pocket costs in connection to their treatment. Individuals can consult a private psychiatrist without a GP referral if they are willing to cover this cost out of pocket.

Hospital admission also requires a GP referral. Individuals are referred to the closest psychiatric department to their home address. If the individual wishes to be referred to another psychiatric department, they can freely choose a hospital with the required experience and capacity, thanks to the free-hospital-choice reform. If this is the case, the individual must pay all travel expenses out-of-pocket.

Specialised care is available in all of the five Danish regions, and community care is located in all of the 98 Danish municipalities. In this study, specialised care refers to psychiatric hospital departments within bigger hospitals. We assume that the departments include psychiatrists. The same assumptions are made for community care institutions. If the waiting time for psychiatric care is longer than defined by national patient rights (30 days or more, depending on the health issue), the individual may freely choose to travel to a farther public provider or to a private provider [27].

The Danish health legislation also states that the healthcare system should act ‘with respect for the individual human being, its integrity and self-determination, and fulfil the need of easy and equal access to healthcare’ (right §2.1 [28]). In this way, equal access in the Danish healthcare system depends on each individual’s needs, and it is therefore important to adjust for each individual’s needs when evaluating variation in access to and utilisation of the healthcare system. A complete needs adjustment is complex. Needs not only depend on the individual’s diagnosis and the severity of their illness, but also on how the individual copes with receiving care, and on characteristics such as age, sex, and socioeconomic status [29]. In this study, we adjust for sociodemographic characteristics, characteristics associated with health status (e.g., diagnosis group, long-term illness, and comorbidity), characteristics associated with the type of care each individual receives (e.g., whether the individual is provided with psychotherapy, as it might be correlated with the individual’s recovery process, and other characteristics, such as whether the individual has been sentenced with a fine or to prison [30]. Criminality combined with a mental disorder is assumed to be a proxy of the complexity of the treatment course [31].

Whether an individual gets a psychiatric hospitalisation depends on the individual’s needs, the treatment the individual has received before the potential hospitalisation and the individual’s disease trajectory. Furthermore, the decision of hospitalisation can also be explained by different aspects of the healthcare system besides the hospitals’ decision on whether the individual should be admitted or not. Importantly, the GP’s role as a gatekeeper affects whether an individual gets more specialised care. Individuals who are treated by different kinds of community-based care tend to be better functioning, and thus receive the care they need when they need it [32]. These treatment alternatives may, therefore, have an indirect effect on psychiatric hospitalisations. In this study, we adjust for characteristics related to each of these institutions to make them comparable.

### 2.2. Study Population and Sampling

This study includes all psychiatric patients aged 18–65 years of age who were treated in the Danish psychiatric sector during 2016. This group is identified as individuals who have been diagnosed with a psychiatric diagnosis based according to chapter F in the ICD-10. The study does not include children and adolescents (0–17 years of age) or the elderly (66+ years of age) because the treatment approach is different for these populations.

### 2.3. Data and Variables

#### 2.3.1. Data

The study is based on combined individual-level data from the following Danish administrative registries: the Central Person Register [33], the National Patient Register [34], the Disability Register [35], the Health Insurance Register [36], the Income Statistics Register [37], the Register of Criminal Statistics [38], the register on personal labour market affiliation [39], and the Danish Education Register [40]. Data on provider characteristics are based on aggregated individual-level data from the listed registers. Additionally, department types are obtained directly from the National Patient Register and the Health Insurance Register.

#### 2.3.2. Outcome: Hospitalisations

The outcome is the individual’s total number of psychiatric inpatient hospitalisations in 2016. This is a broad measure that fits every diagnosis group and also a treatment choice that is influenced by the decisions of different actors. The outcome is log-linearised and standardised to have a mean of zero and a standard deviation of one to enhance normally distributed residuals and numerical stability.

Around 30% of the included individuals were hospitalised in 2016, hence the outcome follows a zero-inflated, left-skewed distribution with a bell-like shape (Figure S1 in Supplementary Materials). Due to this zero-inflated distribution, a hurdle model was constructed for the multiple-membership, multiple-classification model. The hurdle in this model represents whether individuals are hospitalised. The normal distribution and zero-inflation of the outcome are further illustrated in the Supplementary Materials (Figure S2 and Figure S3). The occurrence of hospitalisations is roughly equally distributed over the 12 months of 2016 (Figure S4 in Supplementary Materials).

#### 2.3.3. Characteristics: Using the Andersen Behavioural Model of Utilisation

In our model, individual-level characteristics capture the predisposing and need factors from the Andersen Behavioural Model of Utilisation, while healthcare provider (HCP), including community-based care provider, and healthcare provider municipality (HCPM) characteristics, as well as individual’s municipality characteristics represent enabling factors. The inclusion of various continuous and binary variables allows for a comprehensive exploration of the factors influencing healthcare utilisation.

#### 2.3.4. Individual-Level Characteristics

The following individual-level characteristics are included: sex, defined as whether the individual is male (0/1); age at the time of diagnosis, defined as a continuous variable; whether the individual is a Danish citizen (0/1); whether the individual is married (0/1); whether the individual lives alone (0/1); labour market attachment, defined as studying, working, or receiving unemployment benefits (0/1); income, defined as the individual’s disposable income; whether the individual was sentenced to pay a fine (0/1); whether the individual was sentenced to go to prison (0/1); the diagnosis group the individuals’ diagnosis belongs to (organic disorders (F00–F09), substance abuse (F10–F19), schizophrenia (F20–F29), mood disorders (F30vF39), neurotic disorders (F40–F48), eating disorders (F50), personality disorders (F60), intellectual disabilities (F70–F79) and behavioural disorders (F90–F98)) (0/1); whether the individual was diagnosed with the main diagnosis in 2011 (5 years from 2016) (0/1); psychiatric comorbidity, defined as the number of psychiatric diagnoses other than the main diagnosis; somatic comorbidity, defined as the number of somatic diagnoses; depressive symptoms, defined as whether the individual was diagnosed with depressive symptoms at the time of the main diagnosis (0/1); suicide attempt, defined as whether the individual attempted suicide in 2016 (0/1); number of bed days per hospitalisation, defined as the ratio between the total number of bed days in 2016 and the total number of hospitalisations in the same year; whether the individual has been admitted to forensic psychiatry (0/1), and finally, whether the individual has received psychotherapy at a psychiatric hospital (0/1).

All characteristics were measured for 2016. Crime (whether the individual was sentenced to pay a fine or sentenced to go to prison) is included as a measure of risky behaviour that is associated with psychiatric hospitalisations [41]. Being sentenced to a fine is assumed to be a measure of mild risky behaviour, and being sentenced to prison is assumed to be a measure of severe risky behaviour. The diagnosis groups are defined as in Plana-Ripoll et al. 2019, and classified through the International Classification of Diseases, version 10 (ICD-10) [42]. All diagnosis groups are included because we want to measure all types of individuals in the Danish psychiatry, but as organic disorders, intellectual disorders and developmental disorders are different from the rest of the diagnosis groups (and sometimes not even considered psychiatric diagnoses), we have dropped them from the sensitivity analyses [7,43]. We included whether the individual had the diagnosis 5 years before 2016 as a measure of long-term illness and thus the severity of the diagnosis.

#### 2.3.5. HCP-Level Characteristics

Observed HCP characteristics are divided into hospital department characteristics, GP characteristics, and community-based care provider characteristics. HCP-level characteristics included are the Danish region in which the hospital is located, two dummies for whether the hospital department is inpatient or outpatient, a variable for whether the hospital is a teaching hospital (0/1), and the hospital departments’ yearly bed capacity. GP characteristics include the following continuous variables: number of individuals per GP, number of consultations per GP, number of sessions with talk therapy the GP has provided, and number of e-mail or phone consultations per GP. Unfortunately, we did not have access to any other data on the specific GP. Talk therapy is defined as psychotherapy provided by the GP.

#### 2.3.6. HCPM-Level Characteristics

Observed municipality characteristics include whether the given municipality provides assisted living, personal and practical support, and psychological therapy. Furthermore, continuous variables for the municipality’s population density and the number of Danish individuals are included. These two measures refer to the population of individuals receiving psychiatric care—the study’s population. HCP characteristics and HCPM characteristics are included as weighted averages according to their corresponding multiple membership weights, as further explained below.

### 2.4. Analytical Framework

**Figure 1:**
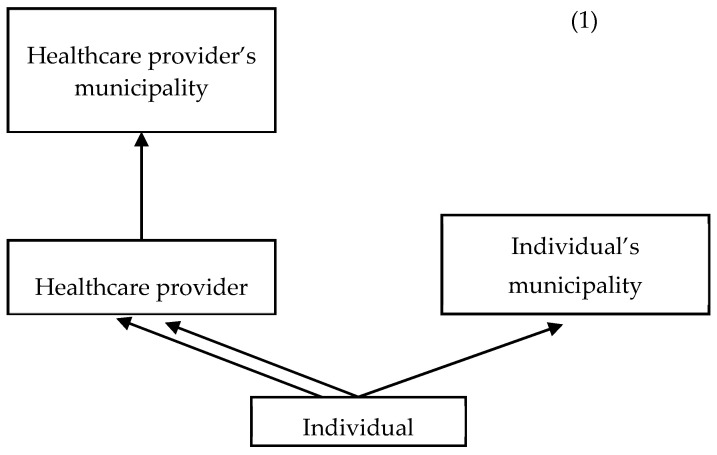

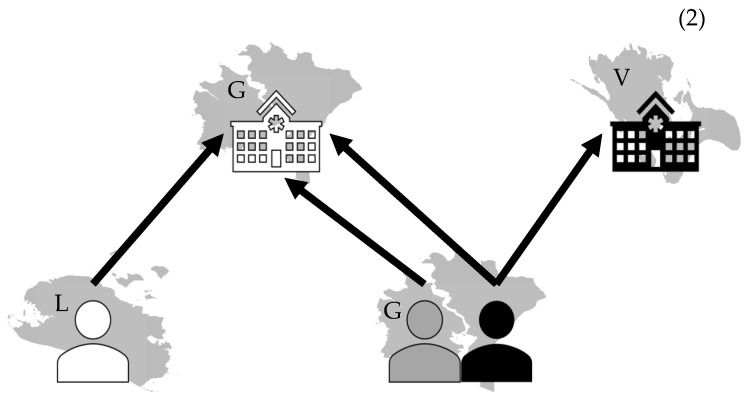
(1) Classification diagram for the MMMC model with an additional hierarchical level. (2) Example of the complex structure of the Danish healthcare system. The diagram shows three different Danish municipalities: Lolland, Guldborgsund, and Vordingborg (L, G, and V, respectively), two different healthcare providers, one located in G and the other one located in V, and three individuals (white, grey, and black). The white individual resides in L and the grey and black individuals reside in G. The three individuals attend the same healthcare provider located in G. Additionally, the black individual attends the second healthcare provider located in V. Source: Created by authors using plotDK for municipality polygons [46].

Nested structures in health and social data are more the norm than the exception [10,44,45]. In this study, individuals are clustered within their place of residence and the HCPs they visit, and the HCPs are clustered within the municipality where they are located (HCPM) (see Figure 1 (1)).

We expect two HCPs located in the same municipality or two individuals who are treated by the same HCP to be more alike than two HCPs that are located in different municipalities. For example, the budgets of HCPs are similar if they are located within the same region [47]. This fact challenges the assumption of independence between observations, which should be fulfilled when estimating single-level linear regression models. One solution is to use multilevel models to examine variation in healthcare. In this type of model, we recognise the complex nesting structures that arise from this type of data. The example given in Figure 1 (2) illustrates that individuals can attend HCPs outside their municipality of residence (cross-classified structure), and that a single individual can attend multiple healthcare providers (a multiple membership structure). In addition to this complex data structure, only around 30% of individuals who visit HCPs are treated with psychiatric hospitalisations, which leads to a distribution of the outcome of interest (number of hospitalisations) with a large proportion of zeros. This section explains how we approach these two particularities from a modelling perspective.

#### 2.4.1. Zero Inflation

To address zero inflation, we estimate a hurdle-like model [48], which is a two-component model. The first component is the probability of not getting a psychiatric hospitalisation, which is modelled as a mixed-effects probit model that acknowledges clustering of individuals within their municipalities. The second component models the (log-transformed) number of hospitalisations for those individuals who have at least one hospitalisation. This implies a data generating process in which HCPs first decide whether the individual should be hospitalised and, if so, the HCP decides how many hospitalisations the individual requires.

This two-part model can be estimated as two independent equations, conditional on the independent variables [49]. These two components were bootstrapped with 100 iterations to estimate standard errors that account for the two-step modelling. An alternative to a hurdle model is a zero-inflated model [50,51,52]. Zero-inflated models assume that there are both sampling and structural reasons for individuals to have zero psychiatric hospitalisations. These models also make specific assumptions about the relationship between the mean and the variance, such as that they are equal (in the case of a zero-inflated Poisson model) or that the variance is a proportion of the mean (in the case of a zero-inflated negative binomial model) [53]. The lack of convergence for models of this type using our data indicates that such assumptions are not held in the case of this paper.

#### 2.4.2. Clustered Structure

As explained above, the data structure is not hierarchically nested. In turn, the data is better described as following a multiple-membership, multiple-classification structure. The multiple-membership component arises because individuals are simultaneously nested within multiple HCPs, with a proportion of their treatment time allocated to each of them. Here, we assume that all HCPs are at the same level, with individuals belonging to more than one HCP and HCP type simultaneously. For example, an individual can visit two different GPs and one hospital. An alternative cross-classified structure would assume that GPs and hospitals, for example, are entirely different structures (like municipalities and HCPs are). We assume that this is not the case, as both GPs and hospitals are care providers.

HCPs are located in their respective unique municipality (with a total of 98 municipalities for all HCPs), which means that there is a hierarchical structure in which HCPs are nested within their municipalities. However, it is necessary to account for an additional source of geographic variation: individuals’ residence municipality. Due to the free hospital choice in Denmark, individuals can attend HCPs that are in a municipality away from where they reside; individuals are cross-classified within their municipality of residence and their HCPs [47]. That is, not all individuals from the same HCP live in the same municipality and not all the residents of a municipality attend the same HCP.

This complex data structure can be modelled using a multiple-membership, multiple-classification (MMMC) model [24], which accounts for the multiple membership of individuals to HCPs, the hierarchical nesting of HCPs within HCPMs, and the cross-classification of individuals within HCPs and their municipality of residence. The model has a fixed part and a random part. In the fixed part, a set of coefficients estimate the relationships between a set of individual, HCP, and HCPM characteristics and the dependent variable. We assume these relationships are fixed across individuals (hence the ‘fixed part’ denomination). The random part of the model includes independent multiple-membership, hierarchical, and cross-classified components described above. We assume that each of these components varies randomly, with a mean of zero and a variance to be estimated. This complex random part implies that the variation in the number of hospitalisations (the dependent variable) changes for every individual according to their HCP and HCPM profile (i.e., the proportion of care time they spend at each HCP and HCPM), but is constant across all individuals residing in the same area.

To help us understand this variation and the role of HCPs and their location, we propose studying a set of scenarios with different care profiles for both HCPs and HCPMs. Then, we calculate the variance partition coefficients for each of the variance components, which show the proportion of the variance in the number of hospitalisations that can be attributed to each of them [54]. This process allows us to understand how important the variation at each of these levels (individuals, HCPs, HCPMs, and individuals’ municipalities of residence) is to explain the variation in psychiatric care.

#### 2.4.3. Model

The proposed model is a two-part model. The first part is the probability of a zero, and the second part is a mean function. These are given by:

(1) $$P\left(y_{i}=0\right)=\Phi \left(Z_{i\;municipality}\alpha +\varepsilon_{municipality}\right),\;y_{i}=0\;\log\;\left(y_{i}\right)=X_{i}\beta +u_{municipality\left(i\right)}^{\left(2\right)}+\underset{jk\;\in \;HCP\left(i\right)\;}{\sum }w_{i,jk}^{\left(3\right)}u_{jk}^{\left(3\right)}+\underset{k\in HCPM\;\left(i\right)}{\sum }\omega_{i,k}^{\left(4\right)}v_{k}^{\left(4\right)}+e_{i},\;y_{i}>0\;\sum_{jk=1}^{JK}w_{i,jk}^{\left(3\right)}=1,\;\forall i\;\sum_{k=1}^{K}\omega_{i,k}^{\left(4\right)}=1,\;\forall i\;\varepsilon_{municipality}\sim \;N\left(0,\;\sigma_{\varepsilon }^{2}\right)\;u_{municipality\left(i\right)}^{\left(2\right)}\sim N\left(0,\;\sigma_{u\left(2\right)}^{2}\right)\;u_{jk}^{\left(3\right)}\sim N\left(0,\;\sigma_{u\left(3\right)}^{2}\right)\;v_{k}^{\left(4\right)}\sim N\left(0,\;\sigma_{v\left(4\right)}^{2}\right)\;e_{i}\sim N\left(0,\;\sigma_{e}^{2}\right)$$

where $y_{i}$ is the number of hospitalisations for individual i and Φ is the standard normal cumulative distribution function. The probability function is a hierarchical probit model with individuals nested within municipalities. $Z_{i\;municipality}\alpha$ is the fixed part that includes the effects of individual characteristics linked to the probability of not being treated with psychiatric hospitalisations.

The mean function has a fixed part $(X_{i}\beta$) and a random part $\left(u_{municipality\left(i\right)}^{\left(2\right)}+\underset{jk\;\in \;HCP\left(i\right)\;}{\sum }w_{i,jk}^{\left(3\right)}u_{jk}^{\left(3\right)}+\underset{k\in HCPM\;\left(i\right)}{\sum }\omega_{i,k}^{\left(4\right)}v_{k}^{\left(4\right)}+e_{i}\right)$. β is a vector of parameters, which we assume are fixed across individuals, associated with the variables in $X_{i}$. The random part of the model includes multiple-membership and cross-classified components, where $e_{i}$, $u_{municipality\left(i\right)}^{\left(2\right)}$, $u_{jk}^{\left(3\right)}$ and $v_{k}^{\left(4\right)}$ are individual, individuals’ municipality, HCP, and HCPM independent random variables that follow a normal distribution with mean zero and variance $\sigma_{e}^{2}$, $\sigma_{u\left(2\right)}^{2}$, $\sigma_{u\left(3\right)}^{2}$ and $\sigma_{v\left(4\right)}^{2}$, respectively.

The multiple-membership component $\underset{jk\;\in \;HCP\left(i\right)\;}{\sum }w_{i,jk}^{\left(3\right)}u_{jk}^{\left(3\right)}+\underset{k\in HCPM\;\left(i\right)}{\sum }\omega_{i,k}^{\left(4\right)}v_{k}^{\left(4\right)}$ accounts for the HCP j that individual i visited, according to the proportion of the individual’s total care spent with that HCP, $w_{i,jk}^{\left(3\right)}$. At the same time, each HCP is nested within the municipality k where it is located, and individuals visit HCPs located in that municipality for a proportion $\omega_{i,k}^{\left(4\right)}$ of their time across all the HCPMs where they received care. The cross-classified component $u_{municipality\left(i\right)}^{\left(2\right)}$ captures the individuals’ municipality of residence, which is not necessarily the same as the HCPM.

The total variance in the number of hospitalisations $y_{i}$ is therefore given by

(2) $$Var\left(y_{i}\right)=\sigma_{u\left(2\right)}^{2}+\sigma_{u\left(3\right)}^{2}\sum_{jk=1}^{JK}{\left(w_{i,jk}^{\left(3\right)}\right)}^{2}+\sigma_{v\left(4\right)}^{2}\sum_{k=1}^{K}{\left(\omega_{i,k}^{\left(4\right)}\right)}^{2}+\sigma_{e}^{2}$$

That is, the total variance in the number of hospitalisations changes for every individual according to their HCP and HCPM profile (i.e., the weighting schemes $\sum_{jk=1}^{JK}{\left(w_{i,jk}^{\left(3\right)}\right)}^{2}$ and $\sum_{k=1}^{K}{\left(\omega_{i,k}^{\left(4\right)}\right)}^{2}$ for each individual) but it is constant across all individuals residing in the same area.

For individuals who only visit one HCP (and therefore one HCPM—10% of all individuals), the total variance simplifies to

$$Var\left(y_{i}\right)=\sigma_{u\left(2\right)}^{2}+\sigma_{u\left(3\right)}^{2}+\sigma_{v\left(4\right)}^{2}+\sigma_{e}^{2}$$

Hence, estimating model (1) allow us to estimate the relative importance of each of these structures in explaining the variability in number of hospitalisations [54].

Random effects are assumed to be normally distributed in multilevel models. We examine this by predicting empirical Bayes estimates of the four different random effects, together with their standard errors, and plotting them. We estimated a Pearson’s correlation matrix to examine the possible correlations between the groupings (see Supplementary Materials).

The models were estimated via maximum likelihood, using the commands *meprobit* and *xtmixed* in STATA 18, and standard errors were estimated via Bootstrap, with 1000 iterations.

### 2.5. Robustness Check and Subgroup Analyses

To test the robustness of our model, we compared the estimation results with alternative specifications and, in the case of nested models, we compared them using likelihood ratio tests. We conducted five different analyses with this aim. First, we evaluated four different models: a MMMC model without the hierarchical component of the HCPM, a cross-classified model with individuals nested within HCPs and their municipality of residence, a multiple membership model only considering HCPs, and a single-level model.

Second, we addressed outliers by re-estimating the main model after excluding extreme cases, defined as individuals with more than 10 hospitalisations on the standardised scale.

Third, we examined the impact of diagnosis diversity by estimating the model without adjusting for diagnosis groups and conducting subgroup analyses. Specifically, we excluded organic disorders, intellectual disabilities, and developmental disorders, and separately analysed individuals with schizophrenia or mood disorders.

Fourth, we explored the influence of individual inclusion times by conducting subgroup analyses excluding individuals newly diagnosed in 2016.

Finally, we conducted a robustness check using the number of inpatient days as the dependent variable. This analysis provided additional insights into healthcare consumption severity and allowed us to compare results with the primary model. Additionally, we controlled for average length of stay to ensure consistency across outcomes.

### 2.6. Ethics

The study is based on national, administrative, third-party data owned by Statistics Denmark. Access to these data can be obtained after individual application by an authorised research group. After approval, pseudo-anonymised data can be accessed at protected servers at Statistics Denmark. The identity of individual persons has not been disclosed to the research group at any point.

## 3. Results

### 3.1. Participants

The study population includes 64,694 individuals, attending 2101 GPs, 146 psychiatric community-based care institutions, 98 municipalities, and 46 psychiatric hospital departments (Table 1). The remaining GPs do not treat or include any individuals with severe mental illnesses.

Around 30% of the individuals were hospitalised at a psychiatric department in 2016. The individuals’ average psychiatric hospitalisation rate was 5.8 hospitalisations, with an average of 8.6 days per hospitalisation (Table 2). A total of 70% of the individuals were treated with treatment types other than hospitalisation, such as outpatient consultations or home visits. A total of 2.7% of the individuals were connected to forensic psychiatry, and 23% were prescribed psychotherapy.

Looking at diagnostic characteristics, 20.5% of the individuals had depressive disorders and 15.5% had lived with their main diagnosis in the past 5 years. The individuals had on average of 1.5 somatic disorders in addition to their psychiatric diagnoses, and a comorbidity rate of 0.5 additional psychiatric disorders. The most common psychiatric diagnosis group in this study population was neurotic disorders, followed by mood disorders. The least common psychiatric diagnosis group was eating disorders. Additionally, we measured three different risky behaviours: 2.7% of the population attempted to commit suicide in 2016; 2.5% committed a crime that was sentenced with prison, and 4.2% committed a crime that was sentenced with a fine. Individuals had a yearly mean disposable income under 150,000 DKK (approximately 38% below national average, calculations available upon request) and almost 20% received a disability pension. Almost 45% were living alone. More than half of the population was female, and the mean age was 36.2 years old.

Twelve percent of the included hospital departments were inpatient departments (Table 2); 26% of all departments were departments at a teaching hospital, and they had a mean bed capacity (the mean number of individuals admitted at one department at the same time) of 26.3 beds per department. We included 2101 GPs who served, on average, 40 individuals with a psychiatric diagnosis each. Furthermore, we included 146 community-based care providers, who were divided into less than 5 outreaching teams, 139 private psychiatrists, and less than 5 psychiatric community departments. Each of them covered, on average, almost 7 individual episodes a day in 2016. Most of the HCPs treating individuals from our population were based in the capital region (35%), and the least were in the Zealandic region (13.12%).

All Danish municipalities were included in this study; 90% of them supplied assisted living for this population; 80% supplied personal and practical support; and 21% supplied therapy provided by a psychologist.

### 3.2. HCP Utilisation Pattern

In 2016, each individual visited an average of 2.23 different HCPs, including GPs, hospitals, and community-based providers, with a median of 2 and a 99th percentile of 8 HCPs. A few individuals visited up to 33 different HCPs (Table 3). Fewer than 20 individuals visited more than 13 different HCPs. These cases were treated as outliers and excluded from the main analysis to ensure the numerical stability of the maximum likelihood estimation algorithm, and to address the impact of extreme outliers. Furthermore, individuals visited up to 12 different HCPMs, and less than 20 individuals visited more than 7 different HCPMs. Cases with more than 7 different HCPMs were excluded as well.

### 3.3. Variation in the Number of Hospitalisations

The hurdle in our model is whether the individual is hospitalised as part of their treatment. Table 4 shows the estimation results for our model for the probability of an individual to be hospitalised in 2016. The model indicates that men, older individuals, Danish citizens, those with specific disorders such as organic, mood, neurotic, and eating disorders, individuals without behavioural disorders, those with substance abuse issues, individuals with a recent history of their disorder, and those who have attempted suicide are more likely to be hospitalised. Additionally, individuals attending teaching hospitals and inpatient departments with a higher bed capacity have a higher probability of being hospitalised.

**Figure 2:**
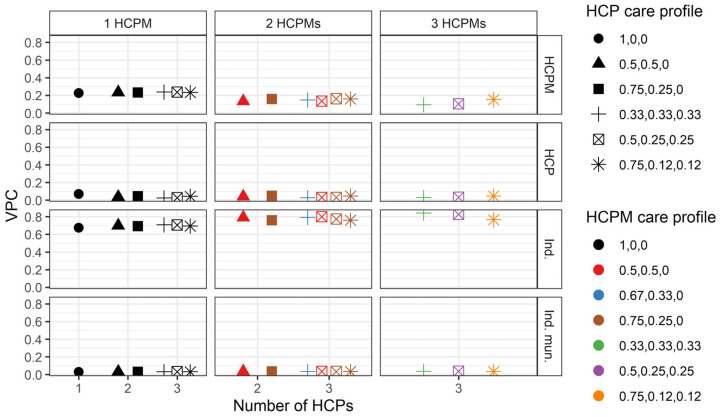
Variance partition coefficients (VPC) for the variance component model for different hypothetical number of healthcare provider and healthcare provider municipality numbers and care profiles. Shapes represent different HCP care profiles $w_{i,\mathrm{jk}}^{\left(3\right)}$ and colours represent different healthcare provider municipality care profiles $\omega_{i,k}^{\left(4\right)}$. The horizontal axis represents the number of healthcare providers, and each vertical subgraph represents a different number of HCPMs. The vertical axis represents the VPC for each of the variance components of the model. HCPM: healthcare provider municipality (4), HCP: healthcare provider (3), Ind.: individual *i*, Ind. mun.: individual’s municipality (2).

Our focus here is on examining the variation in the number of hospitalisations for those individuals that were hospitalised. For individuals who only attended one HCP (and hence only one HCPM), 67.4% of the total variance in the number of hospitalisations corresponds to differences between individuals, 22.6% to differences between HCPMs, 7.02% to differences between HCPs, and 3% to differences between individual’s municipalities (Figure 2). This result is fairly persistent across care profiles and numbers of HCPs and HCPMs, as overall, the variability at the individual and HCPM levels contributes the most to the variability in the total number of hospitalisations.

**Figure 3:**
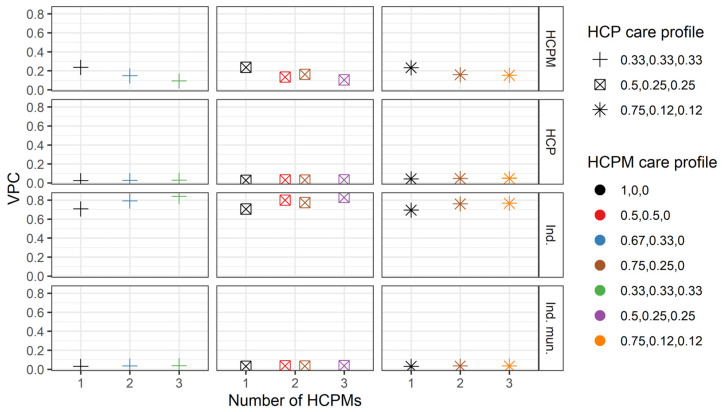
Variance partition coefficients (VPC) for the variance component model for three healthcare providers and different numbers of healthcare provider municipalities and their care profiles. Shapes represent different healthcare provider care profiles $w_{i,jk}^{\left(3\right)}$ and colours represent different healthcare provider municipality care profiles $\omega_{i,k}^{\left(4\right)}$. The horizontal axis represents the number of healthcare providers; the vertical axis represents the variance partition coefficient for each of the variance components of the model. HCPM: healthcare provider municipality (4), HCP: healthcare provider (3), Ind.: individual i, Ind. mun.: individual’s municipality (2).

Figure 2 indicates that deviations from this general pattern are mainly associated with changes in the HCPM care profile. To explore this, Figure 3 focuses on the hypothetical scenario in which individuals attend three different HCPs located in a different number of municipalities and under different care profiles for both HCPs and HCPMs. As shown in the figure, the relative importance of differences between individuals, HCPs, HCPMs, and individual municipalities in the total number of hospitalisations remains stable as the number of HCPMs increases. Furthermore, as Figure 2 shows, the relative importance of differences between individuals increases as the number of HCPs increases. For example, if we compare individuals who attended two and three HCPs who took equal proportions of care (health care profiles 0.5, 0.5, 0 and 0.33, 0.33, 0.33), all in the same HCPM (HCPM care profile 1, 0, 0), 69.9% of the variance in the total number of hospitalisations can be attributed to differences between individuals for those who visited two HCPs, but this increases to 70.7% for individuals that visited three HCPs.

**Figure 4:**
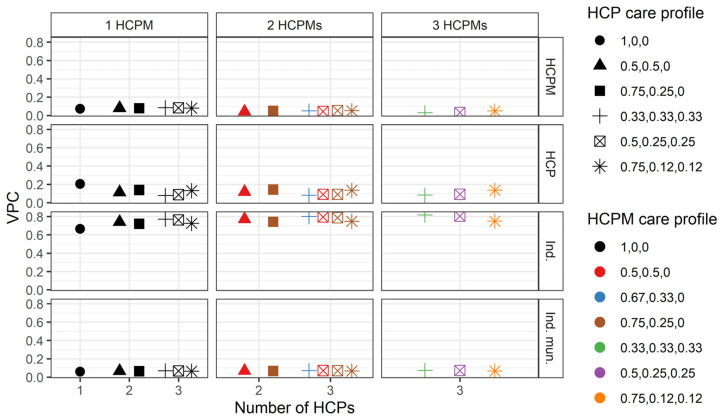
Variance partition coefficients for the model including all characteristics for different hypothetical number of healthcare provider and healthcare provider municipality numbers and care profiles. Shapes represent different healthcare provider care profiles $w_{i,jk}^{\left(3\right)}$ and colours represent different healthcare provider municipality care profiles $\omega_{i,k}^{\left(4\right)}$. The horizontal axis represents the number of HCPs and each vertical subgraph represents a different number of HCPMs. The vertical axis represents the variance partition coefficient for each of the variance components of the model. HCPM: healthcare provider municipality (4), HCP: healthcare provider (3), Ind.: individual i, Ind. mun.: individual’s municipality (2).

When including individual, HCP, and municipality characteristics in the model, these variables explain 68.5% of the variance in the total number of hospitalisations at the HCPM level $\sigma_{v\left(4\right)}^{2}$. Despite the large number of individual-level characteristics, only a very small part of the variance is explained at the other three levels for individuals that only attended one HCP (Figure 4). The relatively small size of the variances at the HCP and individuals’ municipality levels implies that: (a) changes in these variances do not have a substantive meaning, and (b) small decimal approximations during the optimisation process can result in misleadingly large percentual changes when including additional variables in the model. Since the total variance in the number of hospitalisations depends on the care profiles for HCPs and HCPMs, the extent to which these variables explain the total variance in the number of hospitalisations depends on such care profiles.

**Figure 5:**
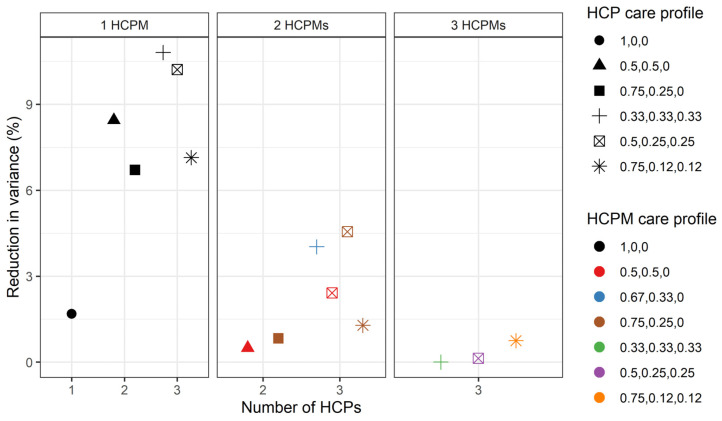
Explained total variance for different hypothetical number of healthcare provider and healthcare provider municipality numbers and care profiles. Shapes represent different healthcare provider care profiles $w_{i,jk}^{\left(3\right)}$ and colours represent different healthcare provider municipality care profiles $\omega_{i,k}^{\left(4\right)}$. The horizontal axis represents the number of HCPs and each vertical subgraph represents a different number of HCPMs. The vertical axis represents the percentage by which the total variance reduced after including all characteristics in the model. HCPM: healthcare provider municipality (4), HCP: healthcare provider (3).

As shown in Figure 5, this implies that the model tends to explain the variance better for individuals who only visited one HCPM than for those who attended two or three HCPMs. In our hypothetical scenarios, the model explains a maximum of 10.8% of the total variance for individuals that attended three HCPs within the same HCPM, with all HCPs providing the same amount of care. In turn, the model does not explain any of the variance for individuals who visited three HCPs in three different HCPMs when care is distributed equally across HCPs, and only explains 0.7% of the total variance for individuals that visited three HCPs in three different municipalities with a 0.75, 0.12, and 0.12 care profile (for both HCP and HCPM).

The increase in the HCPM residual variance component when including individual characteristics indicates that one important aspect in which HCPMs differ between each other is in the characteristics of the individuals they provide care for (Table 5). As an example, Table S1 (Supplementary Materials) shows that individuals with a lower number of bed days per hospitalisation are more likely to receive care in municipalities that offer more personal and practical support options. Furthermore, HCPMs with a lower population density also tend to treat older individuals. In addition, HCP characteristics are correlated with HCPM and individual characteristics. A higher average number of individuals per GP for HCPs is correlated with a higher population density for the HCPM, and HCPs who deliver a larger number of community-based care episodes tend to be located in municipalities with a lower population density (Table S2 in Supplementary Materials). HCPs with a larger bed capacity tend to treat a higher proportion of male individuals, individuals with a higher comorbidity rate, individuals who have been sentenced to prison, and those with a higher rate of bed days per hospitalisation (Table S3 in Supplementary Materials). GPs who treat a lower number of individuals tend to treat younger individuals, individuals not attached to the labour market, individuals with a lower somatic comorbidity rate, and those with a lower number of bed days per hospitalisation.

The fixed part of the full model showed that the number of hospitalisations is larger when the individual is a woman, is older, lives alone, receives a disability pension, has not been sentenced to a fine, has been diagnosed with substance abuse, an organic disorder, a neurotic disorder, a personality disorder, an intellectual disability, a behavioural disorder or a developmental disorder, has been suffering from the disease in the past 5 years, has an increased comorbidity rate and somatic comorbidity rate, has had depressive symptoms, has attempted suicide, has fewer bed days per hospitalization, and is included in forensic psychiatry (Table 5).

The model also shows that a larger number of psychiatric hospitalisations is associated with providers in the northern region and central regions of Denmark, inpatient departments, departments with lower bed capacity, and teaching hospitals (Table 5). A lower number of psychiatric hospitalisations is associated with GP or private psychiatrist HCPs.

### 3.4. Robustness Checks

Our robustness checks for the specification of the random part of the model are shown in Table 6 and in the Supplementary Materials. As expected, all four models show that between-individual variance is the largest variance component of variation in the number of hospitalisations. The robustness checks also show that alternative models that do not account for geographic variation (those that exclude the HCPM or individuals’ municipality components) would wrongly attribute this variation to the HCP level (Table S4 in Supplementary Materials). Likelihood ratio tests show that the four-component MMMC model that we have discussed here has the best fit, but with small differences in the AIC and BIC in comparison with its cross-classified counterpart that would incorrectly assume that individuals are nested within their first HCP.

As a second robustness check, we examined the main model without controlling for extreme cases, without controlling for diagnosis groups, and with the number of inpatient days as the dependent variable (Table 6). Focusing on the case of individuals who attend only one HCP, the variance component model (VCM) that controls for extreme cases has a larger total variance, and it attributes a larger proportion of this total variance to the individual’s municipality and HCP levels. The full model controlling for extreme cases also helps to explain a larger proportion of these variance components than the model presented in the main analysis. However, when diagnosis groups are not controlled for in the full model, the variance attributed to the HCP level is lower. Additionally, when the model includes the number of inpatient days as the dependent variable, almost every level has a larger variance, except for the individual’s municipality level. This model attributes a larger proportion of the total variance (for individuals who attend only one HCP) to the HCPM level than to the individual’s municipality level. Overall, the conclusion about the importance of differences between individuals and geography over those between HCPs are confirmed by all the estimates.

Finally, we considered three subgroups of individuals to assess whether the results are influenced by specific individuals or psychiatric illness history. First, models excluding individuals diagnosed with intellectual disabilities, organic disorders, or developmental disorders attribute a larger proportion of the total variance in the number of psychiatric hospitalisations to the HCP level than the main model, for those who attend only one HCP. Second, results from models including only individuals with schizophrenia or mood disorders are consistent with the main results, although the total variance is higher for this subgroup. Third, models excluding individuals first diagnosed and treated for their disorder in 2016 attribute a higher proportion of the variance in the number of psychiatric hospitalisations to the individual’s municipality and HCP levels.

## 4. Discussion

The aims of this study were to provide a policy-relevant model of variation in psychiatric hospitalisations and to explore factors contributing to this variation within the Danish healthcare system. Our main findings confirm our hypothesis that most of the variation is attributed to demand-side factors, followed by practice context, and least, to supply-side differences.

Our results highlight the significance of demand-side factors, such as individual characteristics and clinical severity, in driving psychiatric hospitalisations. The decision to hospitalise individuals is associated with disorders like mood and eating disorders, longer sickness history, and suicide attempts. Higher hospitalisation rates are also linked to HCPs at teaching hospitals and those with larger bed capacity, indicating the importance of HCP specialisation. Additionally, practice context, including HCP characteristics and geographical location, significantly influences variations in service utilisation.

Despite comprehensive adjustments, much of the variation in psychiatric care utilisation remains unexplained, suggesting potential unwarranted variation driven by factors beyond those captured in our model. Such factors could include physician preferences, HCP employee demographics (e.g., position, education, age, gender, socioeconomic status), and organisational preferences [55,56].

Another contributor to this heterogeneity could be the absence of a clinically meaningful severity measure, due to reliance on register-based data. Including medical adherence data or using survey instruments to assess clinical severity could improve future research.

Additionally, our finding that including individual and HCP characteristics increases the variance between HCPs and HCPMs may be explained by links between geography and demographic composition and individual preference. For example, because older individuals stay in less populated areas and younger ones move to larger municipalities for job or education opportunities [57]. Another explanation may be HCP selectivity, as HCPs with higher capacity tend to treat more severe cases. Future research should explore whether HCPs are selective or individuals choose to live near high-capacity HCPs. Additionally, shared decision-making in Danish healthcare may influence care decisions and contribute to unexplained variation at both the individual and HCP levels.

Our study highlights that, for individuals who attend only one HCP, 67.4% of the variance in hospitalisations is attributed to individual differences. This finding is comparable to Ahammer and Schober’s (2020) finding that 73% of the variance in healthcare expenditure is at the individual level [16,58]. These studies note significant unexplained heterogeneity.

The finding that variation is hard to explain is also supported by Ahammer and Schober, who found that differences between GPs explain a maximum of 4.5% of the total variation in expenditure, which is comparable with our results. The results are comparable even though we include all healthcare providers when examining supply-side variation, while Ahammer and Schober included only GPs. Hospital departments, being closer to hospitalisation decisions, might explain more variation. Additionally, our study allowed individuals to visit multiple HCPs, whereas Ahammer and Schober assigned each individual to a GP annually, ignoring individuals’ mobility.

Modelling psychiatric hospitalisations presents challenges due to the distributional characteristics of the outcome variable. The zero-inflated, left-skewed distribution of the outcome reflects the nature of psychiatric care utilisation, requiring a two-stage approach to address these challenges. This approach accommodated the complex clustered data structure while addressing the zero-inflated nature of the data. Due to this study’s setup, we cannot make any causal interpretations.

To enhance model validity, we log-linearised and standardised the outcome to achieve a mean of zero and a standard deviation of one, reducing skewness and facilitating the application of techniques assuming normally distributed residuals. However, challenges persist due to the substantial variation in outcomes and the numerous levels in the data. Attempts to model individuals with hospitalisations greater than zero using count models were unsuccessful, as these models did not converge.

We considered alternative outcome measures, such as the number of inpatient days per individual or the average length of stay, to mitigate distortions in the results caused by variations in hospitalisation duration [11]. Sensitivity analysis showed no meaningful differences between these outcomes, supporting the robustness of our findings. Another challenge with the selected outcome is that some individuals were not part of the mental healthcare system for the entire year, particularly those newly diagnosed in 2016. This shorter inclusion period likely reduced their hospitalisation rates. To address this, we conducted a subgroup analysis excluding individuals not in the psychiatric registers between 2007 and 2015. After accounting for all characteristics, we found no meaningful differences between the two models.

Our contribution to the literature includes a policy-relevant analysis of a consecutive and non-selected population from Denmark’s entire psychiatric healthcare system. This adds knowledge about where utilisation differences arise in the healthcare system and highlights areas for future research. Furthermore, the use of Danish register data allowed us to simultaneously examine the role of individual characteristics that had not been possible in previous studies.

The implications of our findings underscore the importance of addressing demand-side factors and practice context in healthcare policy and resource allocation decisions. Our study aims to understand systemic mechanisms in healthcare provision, not to critique individual professionals or stigmatise particular diagnosis groups.

Future research should explore the causes of unwarranted variation and integrate additional data sources, such as surveys, to capture factors that are not recorded in data registries. Considering illness severity and building on our MMMC model to produce causal evidence, potentially using an instrumental variable approach [59] are also tasks for future research.

## 5. Conclusions

In conclusion, our study offers valuable insights into the factors driving variation in psychiatric hospitalisations. By highlighting the dominance of demand-side factors and the role of practice context, our findings contribute to informed decision-making in healthcare policy and resource allocation by stating that resources could be used more efficiently if treatment was less heterogenous across HCPMs. Furthermore, our study underscores the potential of using MMMC models in all other fields of healthcare research where we need to adjust for complex data structures with a similar psychiatric treatment pattern or comparable to the Danish healthcare systems.

## Figures and Tables

**Table 1:** Identifiers in the dataset.

Level		*N*
1	Individuals with psychiatric diagnoses	64,694
2	Healthcare providers	2287
	General practice	2100
	Community-based care	145
	Hospital department-based care	42
3	Individuals’ municipalities (area of residence)	98
4	Healthcare providers’ municipalities (local areas)	98

**Table 2:** Individual characteristics, healthcare provider characteristics and municipality characteristics; *n* (%) unless otherwise stated.

		*n* (%)	
Individuals *n* = 64,694	Male	29,396	(45.44)
	Age, mean (SD)	36.186	(13.188)
	Danish citizen	58,624	(90.62)
	Married	12,637	(19.53)
	Living alone	28,923	(44.71)
	Labour market attachment	43,293	(66.92)
	Disability pension	12,813	(19.81)
	Income, mean (SD)	149,271	(435,197)
	Sentenced to prison	1584	(2.45)
	Sentenced to a fine	2734	(4.23)
	Diagnoses		
	Organic disorders	1393	(2.15)
	Substance abuse	6679	(10.32)
	Schizophrenia and psychoses	12,698	(19.63)
	Mood disorders	14,971	(23.14)
	Neurotic disorders	23,827	(36.83)
	Eating disorders	1107	(1.71)
	Personality disorders	7848	(12.13)
	Intellectual disabilities	1351	(2.09)
	Developmental disorders	1404	(2.17)
	Behavioural disorder	5155	(7.97)
	Suffered from disease in past 5 years	10,056	(15.54)
	Psychiatric comorbidity, mean (SD)	0.519	(0.841)
	Somatic comorbidity, mean (SD)	1.493	(2.396)
	Depressive disorders	13,273	(20.52)
	Suicide attempt	1703	(2.63)
	Days per hospitalisation, mean (SD)	8.617	(16.582)
	Forensic psychiatry	1729	(2.67)
	Psychotherapy	14,965	(23.13)
	Psychiatric hospitalisation	19,592	(30.23)
	Number of hospitalisations, mean (SD)	5.764	(9.405)
	Std. number of hospitalisations, mean (SD)	0.000	(1.000)
	Individuals treated for the first time in 2016	3507	(5.42)
Healthcare providers *n* = 2287	Regions		
	Capital	797	(34.85)
	Zealandic	300	(13.12)
	Southern	416	(18.19)
	Central	576	(25.19)
	Northern	198	(8.66)
	Hospital departments	42	
	Inpatient	5	(11.90)
	Outpatient	37	(88.10)
	Hospital characteristics		
	Teaching hospital dep.	11	(26.19)
	Bed capacity, mean (SD)	30.294	(28.855)
	General practitioners	2100	
	GP characteristics		
	Patients per GP, mean (SD)	40.322	(94.691)
	Consultations, mean (SD)	338.759	(539.969)
	Talk therapy, mean (SD)	358.970	(592.511)
	Phone/mail consultations, mean (SD)	20.753	(43.918)
	Community-based care departments		
	Outreaching teams	<5	(<4.00)
	Private psychiatrists	139	(95.86)
	Community	<5	(<4.00)
	Community-based care characteristics		
	Episodes per year, mean (SD)	2554.433	(6988.498)
Local area (municipalities) *n* = 98	Assisted living	88	(89.80)
	Personal and practical support	78	(79.59)
	Therapy by psychologist	21	(21.43)
	Population density, mean (SD)	36,671	(50,033)
	Nationality density, mean (SD)		
	Danish	31,309	(38,580)

**Table 3:** Distribution of the number of unique HCPs and HCPMs visited by individuals in 2016, *n* (%).

Total Number of Different Health Care Providers for Each Individual	Full Population *N* = 64,694	%	Population Excl. Organic, Intelligence and Developmental Disorders *N* = 60,603	%	Population only incl. Schizophrenia and Mood Disorders *N* = 27,104	%
1	6486	(10.03)	6141	(10.13)	2659	(9.81)
2	27,583	(42.64)	25,806	(42.58)	11,000	(40.58)
3	17,214	(26.61)	16,214	(26.75)	7420	(27.38)
4	7709	(11.92)	7194	(11.87)	3396	(12.53)
5	3172	(4.90)	2953	(4.87)	1443	(5.32)
6	1247	(1.93)	1143	(1.89)	610	(2.25)
7	623	(0.96)	562	(0.93)	288	(1.06)
8	276	(0.43)	252	(0.42)	130	(0.48)
9	162	(0.25)	139	(0.23)	70	(0.26)
10	98	(0.15)	93	(0.15)	43	(0.16)
11	55	(0.09)	44	(0.07)	<20	(<0.07)
12	44	(0.07)	40	(0.07)	<20	(<0.07)
13	25	(0.04)	22	(0.04)	<20	(<0.07)
**Total Number of Different Health Care Provider Municipalities for each Individual**	**Full Population** ***N* = 64,694**	**%**	**Population Excl. Organic, Intelligence and Developmental Disorders** ***N* = 60,603**	**%**	**Population only incl. Schizophrenia and Mood Disorders** ***N* = 27,104**	**%**
1	26,347	(40.73)	24,926	(41.13)	11,582	(42.73)
2	26,800	(41.43)	24,950	(41.17)	10,501	(38.74)
3	8664	(13.39)	8089	(13.35)	3687	(13.60)
4	2219	(3.43)	2036	(3.36)	1019	(3.76)
5	527	(0.81)	477	(0.79)	253	(0.93)
6	113	(0.17)	105	(0.17)	49	(0.18)
7	24	(0.04)	20	(0.03)	<20	(<0.07)

Note: Individuals who had visited more than 13 healthcare providers in 2016 are excluded as outliers. Individuals who had visited more than 7 healthcare providers’ municipalities in 2016 are excluded as outliers.

**Table 4:** Results of two-level multilevel probit model for the probability of being hospitalised.

	Dependent Variable: Indicator of Hospitalisation or Not							
		(1) VC model			(2) All characteristics			
		Coef.	SE	Coef.	SE		*p*-val.	
	Male			0.113 **	0.012		0.000	
	Age			0.010 **	0.000		0.000	
	Danish citizen			0.110 **	0.020		0.000	
Diagnosis group	Organic disorders			0.411 **	0.038		0.000	
	Substance abuse			0.937 **	0.020		0.000	
	Mood disorders			0.273 **	0.015		0.000	
	Neurotic disorders			0.074 **	0.014		0.000	
	Eating disorders			0.293 **	0.053		0.000	
	Personality disorders			0.008	0.019		0.667	
	Intellectual disabilities			−0.059	0.042		0.156	
	Behavioural disorder			−0.161 **	0.024		0.000	
	Developmental disorders			0.030	0.041		0.467	
	Suffered from disease in past 5 years			0.541 **	0.016		0.000	
Regions	Suicide attempt			1.070 **	0.036		0.000	
	Capital			5.005	163.997		0.976	
	Zealandic			5.828	163.997		0.972	
	Southern			5.273	163.997		0.974	
	Central			5.382	163.997		0.974	
	Northern			5.256	163.997		0.974	
HCP Characteristics	Teaching hospital			0.593 **	0.038		0.000	
	Outpatient			−1.488 **	0.022		0.000	
	GP			−0.569 **	0.028		0.000	
	Private psychiatrist			−0.689 **	0.081		0.000	
	Outreaching teams			−0.385 **	0.041		0.000	
	Bed capacity			0.021 **	0.000		0.000	
	Patients per GP			0.000	0.000		0.559	
		Var.	SE	Var.		SE		
Levels	Individual’s municipality	0.022	0.004	0.031		0.006		
Observations		64,694		64,694				

Notes: VC: variance components. HCP: healthcare provider. Coef.: coefficient. *p*-val.: *p*-value. ** *p* < 0.01.

**Table 5:** Results of four-component (individual-HCP-HCP municipality-individual municipality) multiple membership multiple classification models.

Dependent Variable: Log-Transformed number of psychiatric hospitalisations in 2016															
		(1) VC Model		(2) Individual Characteristics			(3) HCP Characteristics			(4) Municipality Characteristics			(5) All Characteristics		
		Coef.	SE	Coef.	SE	p-val.	Coef.	SE	p-val.	Coef.	SE	p-val.	Coef.	SE	p-val.
Individual	Male			−0.021 **	0.005	0.000							−0.079 **	0.016	0.000
	Age			−0.001 **	0.000	0.008							0.001 **	0.000	0.001
	Danish citizen			0.005	0.015	0.767							0.032	0.023	0.164
	Married			−0.020	0.013	0.116							−0.018	0.010	0.080
	Living alone			0.039 **	0.012	0.001							0.033 *	0.014	0.016
	Labour market attachment			−0.021	0.022	0.354							0.011	0.006	0.079
	Disability pension			0.219 **	0.032	0.000							0.226 **	0.006	0.000
	Income			0.000	0.000	0.873							0.000 **	0.000	0.000
	Sentenced to prison			−0.007	0.044	0.880							0.007	0.022	0.761
	Sentenced to a fine			−0.100 **	0.006	0.000							−0.075 **	0.014	0.000
	Diagnoses														
	Organic disorders			−0.176 **	0.008	0.000							−0.121 **	0.027	0.000
	Substance abuse			−0.220 **	0.011	0.000							−0.111 **	0.026	0.000
	Schizophrenia and psychoses			Ref.									Ref.		
	Mood disorders			0.108 **	0.010	0.000							0.016	0.016	0.313
	Neurotic disorders			−0.321 **	0.008	0.000							−0.263 **	0.008	0.000
	Eating disorders			−0.084	0.071	0.230							−0.081	0.092	0.379
	Personality disorders			−0.138 **	0.027	0.000							−0.126 **	0.021	0.000
	Intellectual disabilities			−0.157 **	0.030	0.000							−0.131 **	0.010	0.000
	Developmental disorders			−0.171 **	0.017	0.000							−0.146 **	0.042	0.000
	Behavioural disorder			−0.089 **	0.019	0.000							−0.099 **	0.038	0.009
	Suffered from disease in past 5 years			0.202 **	0.020	0.000							0.179 **	0.006	0.000
	Psychiatric comorbidity			0.134 **	0.006	0.000							0.099 **	0.004	0.000
	Somatic comorbidity			0.022 **	0.001	0.000							0.035 **	0.002	0.000
	Depressive disorders			0.162 **	0.007	0.000							0.165 **	0.017	0.000
	Suicide attempt			0.253 **	0.004	0.000							0.204 **	0.011	0.000
	Bed days per hospitalisation			−0.004 **	0.000	0.000							−0.007 **	0.000	0.000
	Forensic psychiatry			0.476 **	0.026	0.000							0.384 **	0.020	0.000
	Psychotherapy			−0.003	0.072	0.410							−0.169 **	0.014	0.000
HCP	Regions														
	Capital			-			−0.132 **	0.035	0.000				−0.080 **	0.007	0.000
	Zealandic			-			−0.214 **	0.039	0.000				−0.214 **	0.018	0.000
	Southern			-			−0.164 **	0.035	0.000				−0.139 **	0.015	0.000
	Central			-			0.922 **	0.050	0.000				0.907 **	0.067	0.000
	Northern			-			0 (omitted)						0 (omitted)		
	Hospital departments			-											
	Inpatient			-			Ref.								
	Outpatient			-			−0.026	0.017	0.116				−0.019 **	0.004	0.000
	Hospital characteristics			-											
	Teaching hospital			-			0.296 **	0.042	0.000				0.273 **	0.042	0.000
	Bed capacity			-			0.000	0.000	0.420				−0.001 **	0.000	0.007
	General practitioners			-			−1.17 **	0.0	0.000				−1.265 **	0.038	0.000
	GP characteristics			-											
	Patients per GP			-			−0.000 **	0.000	0.000				−0.000 **	0.000	0.000
	Consultations			-			Ref.								
	Talk therapy			-			0.000	0.000	0.174				0.000 **	0.000	0.005
	Phone/mail consultations			-			−0.000	0.000	0.945				0.000 *	0.000	0.014
	Community−based care departments														
	Outreaching teams			-			0.017	0.119	0.885				0.111 **	0.015	0.000
	Private psychiatrists			-			−1.242 **	0.047	0.000				−1.121 **	0.042	0.000
	Community			-			Ref.								
	Community-based care characteristics														
	Episodes per year			-			−0.000	0.000	0.434				−0.000 **	0.000	0.002
Municipality	Assisted living			-			-			0.067	0.043	0.256	0.013	0.036	0.711
	Personal and practical support			-			-			0.134 **	0.037	0.000	−0.004	0.004	0.218
	Therapy by psychologist			-			-			−0.175 **	0.021	0.000	−0.017	0.012	0.138
	Population density			-			-			−0.000 **	0.000	0.000	−0.000 **	0.000	0.002
	Nationality density, Danish			-			-			0.000 **	0.000	0.000	0.000 **	0.000	0.002
		Var.	SE	Var.	SE		Var.	SE		Var.	SE		Var.	SE	
Levels	Individual	0.720	0.007	0.773	0.002		0.849	0.005		0.849	0.005		0.699	0.004	
	HCP	0.032	0.018	0.161	0.034		0.178	0.023		0.062	0.017		0.062	0.017	
	HCP’s municipality	0.075	0.009	0.068	0.028		0.088	0.039		0.213	0.020		0.213	0.020	
	Individual’s municipality	0.241	0.036	0.474	0.005		0.450	0.010		0.076	0.007		0.076	0.007	
Observations		19,592		19,592			19,592			19,592			19,592		

Notes: VC: Variance component. HCP: healthcare provider. Coef.: Coefficient. *p*-val.: *p*-value. ** *p* < 0.01, * *p* < 0.5.

**Table 6:** Results from robustness test of four-level (individual-HCP-HCP municipality-individual municipality) multiple membership multiple classification models.

	Dependent Variable: Number of Psychiatric Hospitalisations in 2016																			
	Controlling for extreme cases					Not adjusting for diagnoses				Excl. intellectual disabilities, organic and developmental disorders						Incl. only schizophrenia and mood disorders				
	VCM		Model with all controls			VCM		Model with all controls		VCM			Model with all controls			VCM			Model with all controls	
	Variance	SE	Variance		SE	Variance	SE	Variance	SE	Variance		SE	Variance	SE		Variance	SE		Variance	SE
Individual	0.832	0.004	0.689		0.001	0.720	0.007	0.711	0.005	0.851		0.004	0.675	0.003		0.847	0.003		0.714	0.006
HCP	0.178	0.010	0.073		0.003	0.032	0.018	0.044	0.012	0.488		0.007	0.037	0.324		0.099	0.012		0.000	0.000
HCPM	0.081	0.042	0.218		0.158	0.075	0.009	0.220	0.021	0.087		0.033	0.287	0.072		0.157	0.022		0.328	0.038
Individual’s municipality	0.475	0.011	0.005		0.075	0.241	0.036	0.077	0.005	0.489		0.007	0.080	0.005		0.494	0.010		0.099	0.010
Observations	19,592					19,592				18,230						11,831				
	Excl. individuals treated for the first time in 2016							Number of inpatient days as the dependent variable												
	VCM			Model with all controls				VCM							Model with all controls					
	Variance		SE	Variance		SE		Variance			SE				Variance			SE		
Individual	0.848		0.005	0.710		0.003		0.973			0.009				0.770			0.001		
HCP	0.186		0.041	0.072		0.016		0.157			0.004				0.064			0.014		
HCPM	0.081		0.018	0.170		0.023		0.213			0.034				0.304			0.013		
Individual’s municipality	0.488		0.010	0.078		0.018		0.148			0.007				0.066			0.005		
Observations	19,190							19,947												

Notes: HCP: Healthcare provider, HCPM: Healthcare provider’s municipality. Fixed part estimates not included for conciseness.

## Data Availability

The corresponding author had full access to the data in the study and had final responsibility for the decision to submit for publication. Data were secured within the servers of Statistics Denmark and analysed via a safe password-protected online data access. All data were fully pseudonymised.

## Supplementary Materials

The following supporting information can be downloaded at: https://www.mdpi.com/article/10.3390/ijerph21080973/s1: Figure S1: Normality plot of the distribution of the data using three different models. HCP: Healthcare providers, HCPM: Healthcare provider’s municipality. MM 1: multiple membership for HCP, MM 2: multiple membership for HCP and HCPM, MMC: multiple-membership, multiple-classification; multiple membership for HCP and HCP, and cross-classified for the indi-vidual’s municipality. n = 64,694. Figure S2: Normality qnorm plot of the distribution of the data based on the main model. Figure S3: Density plot of the number of psychiatric admissions on Danish hospitalisations in 2016. Figure S4: Distribution of psychiatric hospitalisations on Danish hospitals in 2016. Table S1. Pearson correlation matrix: Aggregated individual characteristics on the healthcare provider level. Table S2. Pearson correlation matrix: Aggregated individual characteristics on the healthcare provider local area level. Table S3. Pearson correlation matrix: Aggregated healthcare provider characteristics on the healthcare provider local area level. Table S4. Likelihood ratio test on the variance component model.

## List of Abbreviations

HCP	healthcare provider
HCPM	healthcare provider municipality
MMMC	multiple-membership, multiple-classification
GP	general practitioner
OECD	Organisation for Economic Co-operation and Development
VCM	variance component model
Coef.	coefficient
Var.	variance
SE	standard error
