# Supplementary material for: Variation in Psychiatric Hospitalisations: A Multiple-Membership Multiple-Classification Analysis
Source: Int J Environ Res Public Health. 2024 Jul 25;21(8):973. doi: 10.3390/ijerph21080973 (PMC11353323; doi:10.3390/ijerph21080973)
Supplement: Supplementary file 1 [file ijerph-21-00973-s001.zip › ijerph-3072647-supplementary.pdf]

Supplementary Materials

1. Normality Plot 2

2. Normality plot: Qnorm plot 2

3. Density plot: Number of psychiatric admissions 3

4. Distribution of psychiatric hospitalisations in 2016 3

5. Correlations between different level characteristics 4

6. Likelihood ratio test 4

1. Normality Plot

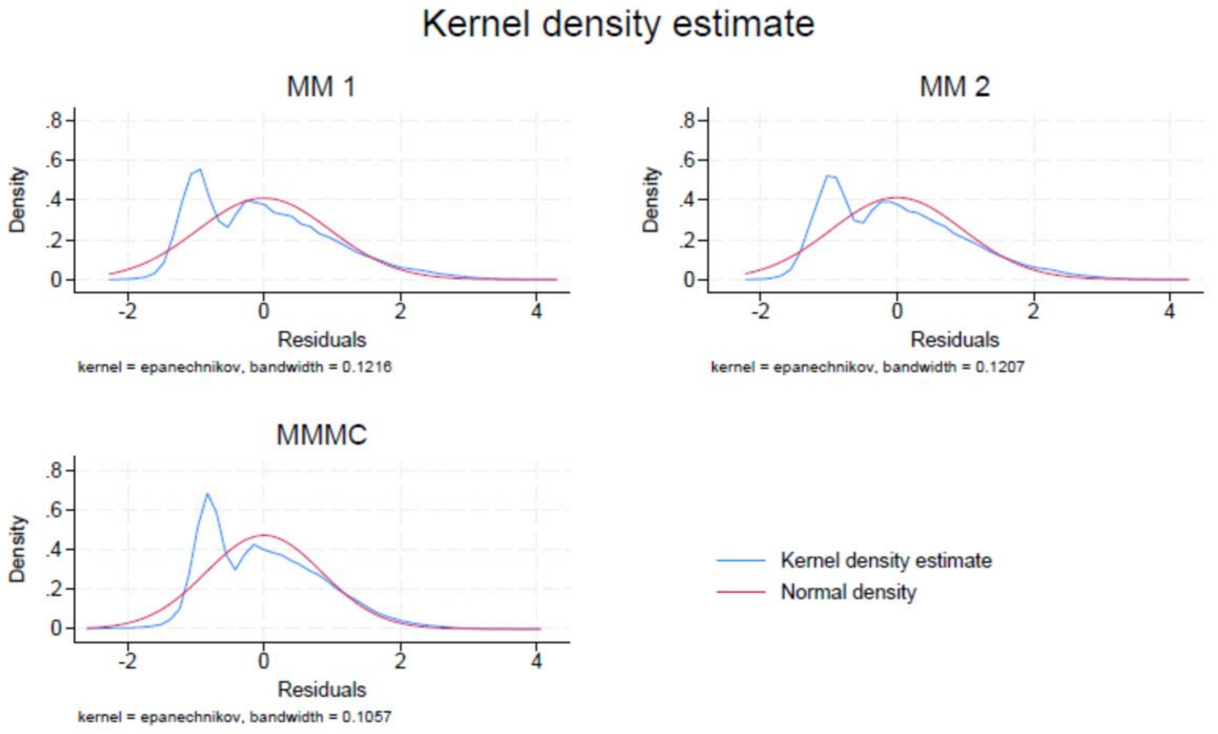

Figure S1. Normality plot of the distribution of the data using three different models. HCP: Healthcare providers, HCPM: Healthcare provider's municipality. MM 1: multiple membership for HCP, MM 2: multiple membership for HCP and HCPM, MMC: multiple-membership, multiple-classification; multiple membership for HCP and HCP, and cross-classified for the individual's municipality. n= 64,694.

2. Normality plot: Qnorm plot

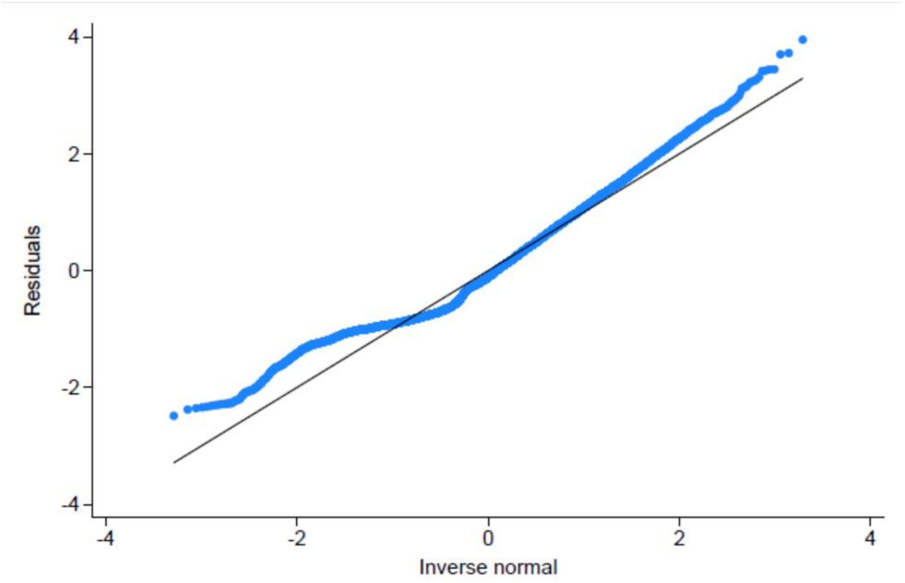

Figure S2. Normality qnorm plot of the distribution of the data based on the main model.

3. Density plot: Number of psychiatric admissions

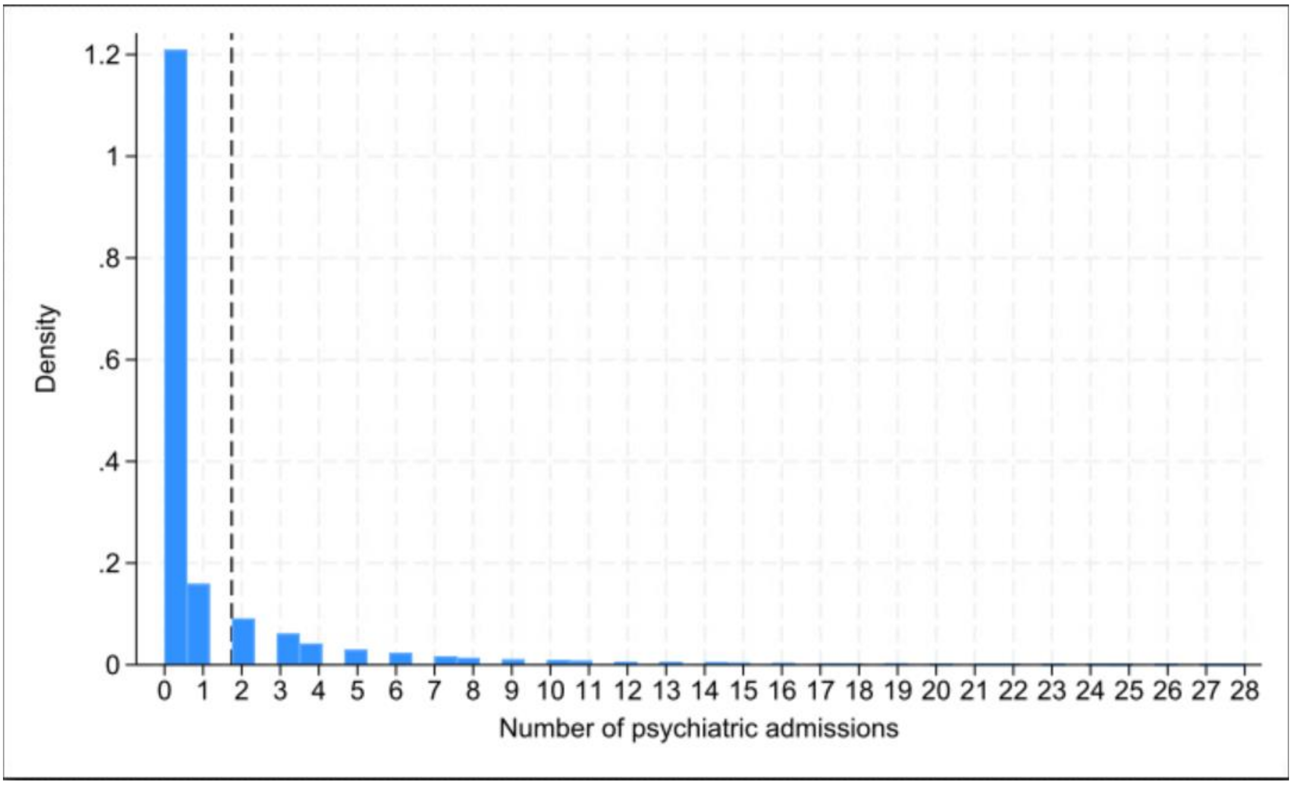

Figure S3. Density plot of the number of psychiatric admissions on Danish hospitalisations in 2016.

4. Distribution of psychiatric hospitalisations in 2016

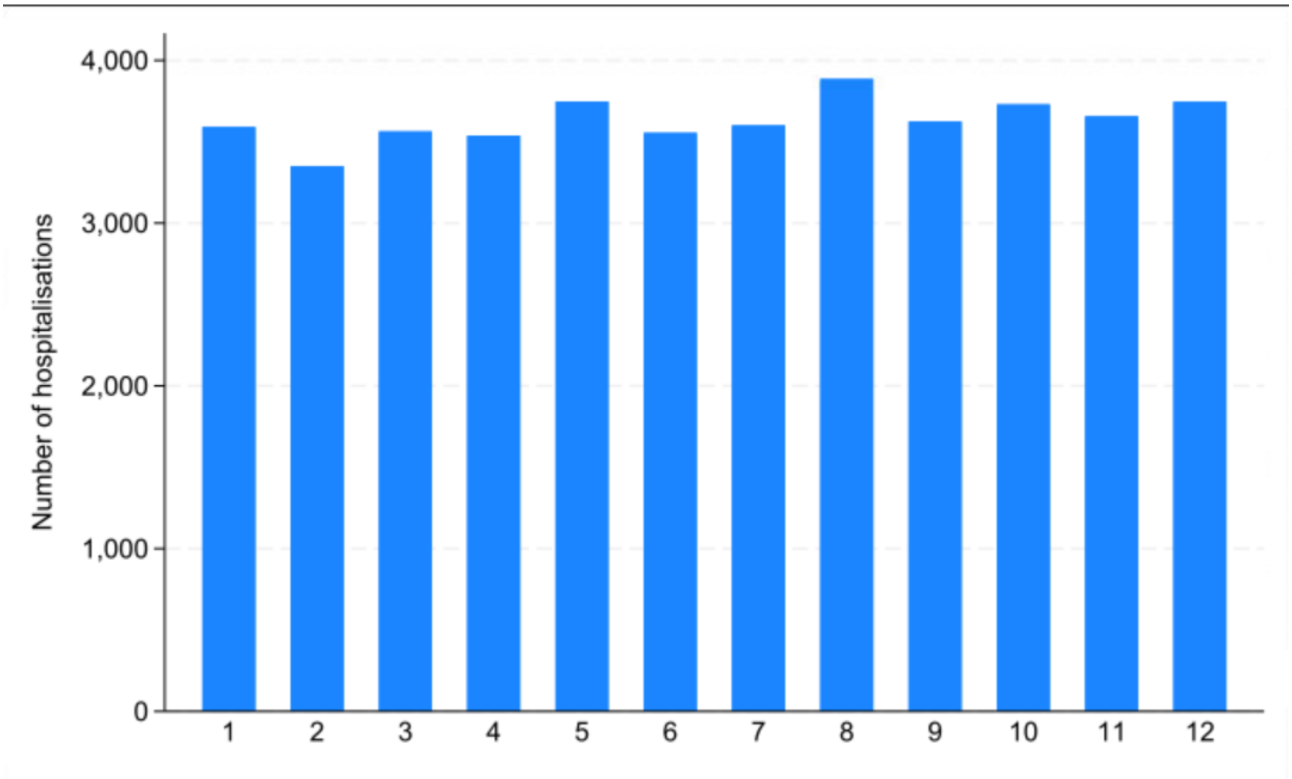

Figure S4. Distribution of psychiatric hospitalisations on Danish hospitals in 2016.

5. Correlations between different level characteristics

Table S1. Pearson correlation matrix: Aggregated individual characteristics on the healthcare provider local area level.

	**1**	**2**	**3**	**4**	**5**	**6**	**7**	**8**	**9**	**10**	**11**	**12**	**13**
1. Population density	1.000												
2. Danish density	0.992*	1.000											
3. Personal and practical support	-0.023	-0.035	1.000										
4. Therapy by psychologist	-0.073	-0.056	0.197	1.000									
5. Male	0.113	0.148	0.118	0.073	1.000								
6. Age	-0.371*	-0.387*	0.152	0.050	0.136	1.000							
7. Danish citizen	0.030	0.048	0.029	0.053	0.055	-0.056	1.000						
8. Income	-0.162	-0.208*	0.091	0.058	-0.207*	0.520*	-0.051	1.000					
9. Labour market attachment	0.148	0.113	-0.155	-0.175	-0.472*	-0.355*	-0.416*	0.167	1.000				
10. Comorbidity	0.141	0.158	-0.058	-0.110	0.131	-0.001	0.300*	-0.145	-0.287*	1.000			
11. Somatic comorbidity	-0.077	-0.104	-0.183	0.102	-0.103	0.047	0.037	0.125	0.006	-0.065	1.000		
12. Sentenced to prison	0.177	0.209*	0.045	-0.099	0.353*	-0.212*	0.160	-0.439*	-0.272	0.324*	0.113	1.000	
13. Bed days per admission	-0.180	-0.210*	-0.227*	0.040	-0.035	0.230*	-0.174	0.301*	0.083	-0.211*	0.423*	-0.190*	1.000

Note: *p-value < 0.05

Table S2. Pearson correlation matrix: Aggregated healthcare provider characteristics on the healthcare provider local area level.

	1	2	3	4	5	6	7	8	9	10
1. Population density	1.000									
2. Danish density	0.992*	1.000								
3. Personal and practical support	-0.022	-0.034	1.000							
4. Therapy by psychologist	-0.075	-0.058	0.200	1.000						
5. Teaching hospital	0.393	0.429*	-0.036	0.092	1.000					
6. Outpatient dep.	-0.069	-0.045	0.008	0.139	0.110	1.000				
7. Community dep.	-0.160	-0.110	-0.095	-0.409	-0.595*	-0.129	1.000			
8. Bed capacity	-0.167	-0.190	-0.062	-0.270	-0.276	-0.442*	0.046	1.000		
9. Patients per GP	0.337*	0.370*	0.013	-0.103	0.985*	0.090	-0.579*	-0.2149	1.000	
10. Community-based episodes	-0.519*	-0.540*	0.130	-0.095	-0.414	-0.038	0.004	0.750*	-0.375	1.000

Note: *p-value < 0.05

Table S3. Pearson correlation matrix: Aggregated individual characteristics on the healthcare provider level.

	**1**	**2**	**3**	**4**	**5**	**6**	**7**	**8**	**9**	**10**	**11**	**12**
1. Bed capacity	1.000											
2. Patients per GP	-0.060*	1.000										
3. Community-based episodes	0.740*	-0.101*	1.000									
4. Male	0.066*	-0.027	0.044*	1.000								
5. Age	-0.032	-0.099*	-0.019	0.152*	1.000							
6. Danish citizen	0.001	0.037	-0.007	-0.045*	-0.004	1.000						
7. Income	-0.022	-0.040	-0.008	-0.028	0.228*	0.062*	1.000					
8. Labour market attachment	-0.019	-0.047*	0.025	-0.105*	-0.368*	-0.125*	0.009	1.000				
9. Comorbidity	0.101*	0.065*	0.031	0.117*	-0.053*	0.027	-0.074*	-0.182*	1.000			
10. Somatic comorbidity	0.010	-0.196*	-0.020	0.030	0.177*	0.005	0.018	-0.125*	0.042*	1.000		
11. Sentenced to prison	0.058*	0.021	0.000	0.209*	-0.045*	-0.000	-0.070*	-0.097*	0.254*	0.016	1.000	
12. Bed days per admission	0.052*	-0.252*	0.021	-0.055*	0.043*	-0.027	0.040	0.056*	-0.112*	0.161*	-0.043*	1.000

Note: *p-value < 0.05

6. Likelihood ratio test

Table S4. Likelihood ratio test on the variance component model.

	**Dependent variable: Log number of psychiatric hospitalisations in 2016**							
	**MM 1 model**		**CC model**		**MMMC model**		**MMMC model**	
	Var.	SE	Var.	SE	Var.	SE	Var.	SE
Individual	0.949	0.009	0.718	0.007	0.721	0.007	0.721	0.036
HCP	0.284	0.138	0.022	0.003	0.039	0.021	0.032	0.018
HCP's municipality	-		0.252	0.038			0.007	0.009
Individual's municipality	-		-		0.240	0.036	0.241	0.036
LR (-2* loglikelihood)	5022.538**		-280.458		6.018*		-	
AIC	54409.81		49669.74		49395.30		49391.28	
BIC	54433.45		49701.25		49426.81		49430.67	

Notes: HCP: Healthcare provider. MM: multiple membership, CC: cross-classified, MMC: multiple-membership, multiple-classification. n= 64,694, AIC: The Akaike information criterion, BIC: The Bayesian information criterion.
